# Employing DNA metabarcoding to determine the geographical origin of honey

**DOI:** 10.1016/j.heliyon.2020.e05596

**Published:** 2020-11-25

**Authors:** Elmira Khansaritoreh, Yasaman Salmaki, Elias Ramezani, Tayebeh Akbari Azirani, Alexander Keller, Katrin Neumann, Kamaleddin Alizadeh, Shahin Zarre, Gudrun Beckh, Hermann Behling

**Affiliations:** a University of Goettingen, Department of Palynology and Climate Dynamics, Untere Karspüle 2, 37073, Goettingen, Germany; b Department of Plant Science, Center of Excellence in Phylogeny, School of Biology, College of Science, University of Tehran, P.O. Box 14155-6455, Tehran, Iran; c Department of Forestry, Faculty of Natural Resources, Urmia University, Urmia, Iran; d Department of Physical Geography, School of Earth Sciences, Shahid Beheshti University (S.B.U), Tehran, Iran; e Center for Computational and Theoretical Biology & Department of Bioinformatics, University of Würzburg, Am Hubland, 97074 Würzburg, Germany; f Lifeprint GmbH, Industriestrasse 12, 89257, Illertissen, Germany; g Quality Service International GmbH, Flughafendamm 9, 28199, Bremen, Germany

**Keywords:** Food science, Food analysis, Food technology, Molecular biology, Honey, Geographical origin, Metabarcoding, ITS2, *rbcL*

## Abstract

Unfavourable climatic conditions force Iranian beekeepers to translocate over large distances in the course of the year. However, irrespective of the main place of production, the honey is always labeled with the name of the beekeepers' hometown, which leads consequently to mislabeled products. The present study investigates the capability of DNA metabarcoding to locate the geographical origin of honey. The molecular markers (ITS2 and *rbcL*) allowed identification of 926 plant species in studied samples. A comprehensive review of floristic reference books specified 34 key species that could be used to successfully determine the geographical origin in 91.4% of samples. These key species were usually present in honey with tiny amounts and thus, conventional palynology might not be able to detect them. The present investigation indicates that although ITS2 is able to detect more species than *rbcL*, utilizing a combination of both markers provides more robust evidence of geographical origin.

## 1 Introduction

According to a 2011 report by Agricultural Bank of Iran, about 60% of Iranian honey is produced in four provinces, including Ardabil, Isfahan, East Azerbaijan and West Azerbaijan, which are situated in the Irano-Anatolian province (Zohary, 1973) of the Irano-Turanian floristic region. The majority of beehives are scattered in areas of these provinces with an average annual precipitation of less than 350 mm. This precipitation mainly occurs in winter or early spring, when temperatures are still low ranging between -1.7 and 4.2 °C. Winters are extremely long and cold and summers are hot and dry (Meteorological Organization of Iran, 2018). Under these climatic conditions plants cannot properly bloom and therefore the flowering season is short and nectar production is poor. Consequently, beekeepers have one of two approaches to tackle this challenge: either, feeding supplementary sugar to bees during the foraging season or by migration to other areas with more favorable climatic conditions. The beekeepers of colder cities migrate to warmer areas in early autumn (October) and may stay at destination until mid-spring (April) to benefit from a longer production season and to promote colony increase. During the late spring (June) and summer, many beekeepers migrate to montane rangelands where the temperature is moderate and bees can visit wildflowers. The beekeepers usually return to their hometowns only to harvest and sell the product. However, even if the honey completely originates from the flora of somewhere else, the name of the beekeepers' hometown is printed on the jar. Meaning that the honey is labeled as originating from a different geographical origin to where it was produced. The reason for this is the nationwide popularity of honey from particular cities. The present study aims to address this issue by providing tools to identify the actual geographical origin of the honey.

There are two methods available to determine the origin of honey based on its pollen content: the conventional palynology approach and the modern DNA based techniques such as metabarcoding. For the present study, DNA metabarcoding is superior to conventional palynology because the latter is often not able to identify pollen types down to species level (Khansari et al., 2012; Rahl, 2008; Salmaki et al., 2008). However, species-level resolution is necessary to identify locally distributed and endemic plants that play a key role in the determination of geographical origin of honey.

During the recent years, metabarcoding has successfully been employed to address variety of research topics such as pollen load analysis and foraging behavior of bees (Danner et al., 2017; De Vere et al., 2017; Hawkins et al., 2015; Kaluza et al., 2017; Nürnberger et al., 2019; Richardson et al., 2015; Wilson et al., 2010), as well as determination of botanical (Bruni et al., 2015; Hawkins et al., 2015; Indo et al., 2019; Laha et al., 2017; Manivanan, 2018; Prosser and Hebert, 2017; Saravanan et al., 2019; Utzeri et al., 2018a, Utzeri et al., 2018b) and entomological (Kek et al., 2017; Prosser and Hebert, 2017; Soares et al., 2018; Utzeri et al., 2018a, Utzeri et al., 2018b; Zhang et al., 2019) origins of honey. We benefited from knowledge gained by these researches to design the current study that focuses on exploring the geographical origin of honey, an aspect that has been missing or less considered in previous investigations. Therefore, while determining the botanical origin involves making an inventory of the most abundant pollen types, this study concentrates on discovering the key species that represent the flore of a specific area.

The authentification of geographical origin is of special importance in honey market because honey is one of the most globally traded food commodities that is subject to different types of adulteration. Having a bad reputation for fraud, some countries are banned from exporting honey to European Union. In the United States of America although more relax regulations are applied regarding the honey import, U.S. Food and Drug Administration severely controls contamination by some sort of antibiotics used by beekeepers in Asian countries. Hence, the authenticity of geographical origin has been extensively investigated by food scientists in recent years. In particular food chemists have suggested several methods to determine the geographical origin of honey in global scale (Wang and Li, 2011; Zhou et al., 2018). In the present study, the capability of DNA metabarcoding to locate the real geographical origin is tested in large scale. Although all of our samples were from one country, the diverse natural and agricultural landscapes visited by Iranian beekeepers, makes it possible to simulate a variety of geographical origins.

## 2 Material and methods

### 2.1 Sampling

A total of 196 honey samples were collected in a 10-day field work from Iranian provinces of Ardabil, Isfahan, East Azerbaijan and West Azerbaijan, of which 70 samples were selected for metabarcoding. The criteria for selection of samples was migration of the beekeeper, preferably to destinations outside the home province. All samples were collected (in plastic jar) from standard wooden beehives of *Apis Mellifera* by cutting the honey comb with knife. After the fieldwork, the samples were shipped to Bremen, Germany and stored at 4 °C in Quality Service International GmbH (QSI) in Bremen. Table 1 summarizes the information about the collected samples.

**Table 1:** Allocation of samples to different places of collection and routes of migration (as reported by beekeepers).

Site of collection	Province	Ardabil		E. Azerbaijan			Isfahan		W. Azerbaijan	
	City	Khalkhal	Sarein	Maragheh	Marand	Sarab	Khansar	Semirom	Khoy	
		14	7	15	2	3	14	6	9	Number of samples
Route of migration	North	13	7			2				
	South/South-west	1		5			13	6	1	
	West			10	2	1	1		8	

**Figure 1:**
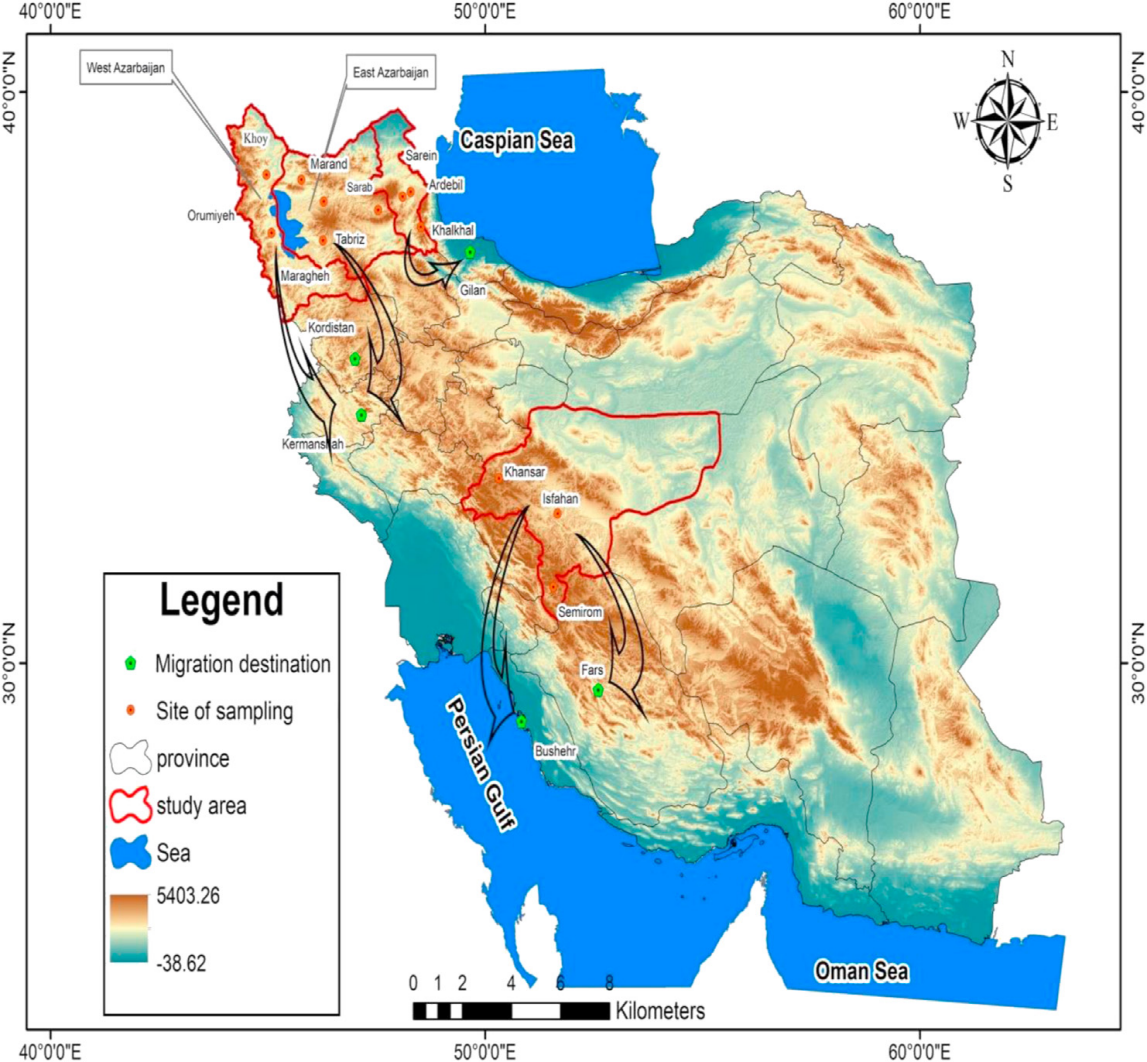
Map of Iran showing the sampling sites and the route of migration between provinces.

According to the information gathered from migrating beekeepers whose honeys were analyzed in the present study, there are three main routes that they take depending on the location of their hometown (Figure 1). The beekeepers of East and West Azerbaijan mainly spend the summer in montane rangelands of western provinces such as Kurdestan and Kermanshah. These rangelands lie within the Zagros floristic zone and have moderate summer temperature and rich floral resources. The beekeepers of Ardabil stay in Guilan from autumn (October) to early spring (March). Gilan is one of the northern provinces of Iran that is situated in the Hyrcanian floristic province and has moderate temperatures during the winter (Takhtajan, 1986). The beekeepers of Isfahan also migrate to warmer areas during the cold seasons, but they take their hives to the south and south-west in particular provinces Fars and Bushehr that are located in central Irano-Turanian and Saharo-Sindian floristic regions respectively (Sagheb-Talebi et al., 2014). The vegetation in each of these areas is characterized by some locally distributed and endemic plant taxa and specific agricultural crops which we consider as the key plants for determination of geographical origin.

### 2.2 DNA extraction and amplification

Prior to DNA extraction, 20 g of each honey sample (extracted by squeezing the comb) was washed with distilled water and centrifuged (on a Hettich Rotina 46) at 1000 g, for 10 min to isolate the pollen grains from other components in honey. This method yielded visible pollen pellets at the conical end of centrifuge tubes in all samples. The DNA extraction was done using NucleoSpin Food Prep Kit from Macherey-Nagel (Düren, Germany) as explained in the product instruction manual.

Amplification was performed for two target regions ITS2 and *rbcL*. The sequences of the standard plant barcoding primers for ITS2 (Chen et al., 2010; White et al., 1990) and *rbcL* (Erickson et al., 2017) were modified to fit the dual-indexing metabarcoding strategy (Sickel et al., 2015). PCR was performed in two separate 25 μl reactions for each sample in order to reduce PCR bias. In addition to the test samples, a negative control, as well as a positive control with a known mixture of species, were included in the run. The PCR mixture contained 12.5 μl AccuStart II PCR ToughMix (Quantabio), 1.25 μl 20x EvaGreen (Biotium), 0.75 μl of each *rbcL* primers (10μM, biomers), 1.25 μl of each ITS2 primer (10μM, biomers), 2.25 μl PCR grade water and 5 μl DNA (about 20 ng/μl). With the dual indexing primers used in this step, each sample was labeled by a different forward/reverse index combination for sample-specific labeling. PCR was conducted on an Applied Biosystems 7300 Real-Time PCR System. Conditions were as follows: initial denaturation at 95 °C for 10 min, 35 cycles of denaturation at 95 °C for 45 s, annealing at 52 °C for 60 s and elongation at 72 °C for 60 s; followed by a final extension step at 72 °C for 10 min. Afterward, a dissociation curve was used as the control for amplification.

### 2.3 Sequencing

After PCR, the duplicates of each sample were combined and purified with magnetic beads (AMPure XP, Beckmann Coulter) by adding 40 μl AMPure beads to 50 μl of the mixed PCR product. After washing twice with 80% ethanol, DNA was eluted in 30 μl of 10 μM Tris (pH 8.5) and the concentration of the mixture was measured using a Qubit fluorometer. All samples were pooled in equal concentrations for the 4 nM library. Next, this library was diluted to 7 pM, denatured and spiked with 15% of the denatured PhiX Control (Illumina) as described in the 16S Metagenomic Sequencing Library Preparation workflow (Illumina). Sequencing was performed on the Illumina MiSeq using 2 × 250 cycles v2 chemistry as described in Sickel et al. (2015).

### 2.4 Data analysis

From all reads obtained, subsets for the two markers ITS2 and *rbc*L were split up by using BLASTn to assess the first ten basepairs of each read, which are conserved for each marker and thus a good discriminator. Then, for each marker, forward and reverse reads were quality filtered, dereplicated, denoised and finally merged to amplicon sequence variants (ASVs) using the DADA2 pipeline (Callahan et al., 2016a, Callahan et al., 2016b). The parameters for quality filtering were set according to the ITS pipeline (Callahan et al., 2016a, Callahan et al., 2016b) and pipeline for *rbc*L diatoms (Keck et al., 2019). The below parameters were set with the same values for both markers:

maxN = 0, maxEE = c(2, 2), truncQ = 2, rm.phix = TRUE

As reads belonging to ITS2 show natural length variation between 200-600 base pairs (bp), in order to obtain the maximum sequence diversity, the truncation was not applied to them, instead minimum accepted length of sequences (minLen) was set to 50. The forward and reverse reads from *rbcL* were truncated at 250 and 170, respectively. These numbers were gained from a quality profile plot. ASVs were taxonomically classified by BLASTn against the nr/nt databases of Genbank (Benson et al., 2009). The order of criteria for selection was minimum E-value = 1e-50, maximum identity = 99%, and coverage = 99%. If more than one hit has the same quality metrics, the one with the taxon that has higher frequency among the hits for that sequence was selected.

### 2.5 Key species selection

All assigned taxa (including other possible taxa) were checked in reference books on the flora of Iran (Barneby et al., 1965; Komarow, 1933; Podlech and Zarre, 2013; Rechinger, 1963; Townsend and Guest, 1965), to find the key taxa that are endemic or characteristic for one of the west, north or south/south-west regions of Iran. Then, the result for each sample was checked for key taxa to determine the possible routes of migration for the producer of that sample.

## 3 Results and discussion

### 3.1 Sequencing

MiSeq delivered 5,377,352 reads in total and approximately 76820 reads per sample on average. Overall, 99.77% of reads were classifiable to markers, with 84.7% ITS2 and 15.07% *rbcL*.

The separated reads were entered the DADA2 pipeline to obtain unique sequences (ASVs) that were submitted to BLASTn for taxonomy assignment. The percentage of reads remained after each step of DADA2 and the number of assigned species are given for each marker in Table 2.

**Table 2:** Number of reads remained after each step of the DADA2 pipeline. The columns that are presented in percentage are calculated out of input reads. ASV: Amplicon Sequence Variant.

Marker	Input	Filtered	Denoised Forward	Denoised reverse	Merged	Non-chimera	ASVs	Species
ITS2	4,577,202	98.5%	98.1%	98.1%	94.4%	78%	1790	674
*rbcL*	788,320	99.6%	99.2%	99.3%	96.6%	83.9%	545	386

### 3.2 Key species

According to available resources on the flora of Iran, only 53% of species found by metabarcoding have been recorded as being present in Iran. Several reasons may underlie this inconsistency. First, compared to Europe, North America and many other well documented regions there are relatively few records of Iranian plants on the Genebank. Therefore, the taxonomic name of non-Iranian close relatives might be assigned to some sequences. Second, during the migration beekeepers might pass through places where the detailed floristic investigation is not yet performed. Thus, it is quite possible that a plant found by metabarcoding is missing on floristic references. Third, all available algorithms used in bioinformatics have some degree of inaccuracy that might cause misidentification of taxa.

Among the species which exist in the flora of Iran, 34 key species were found that correctly matched the migration routes of 64 (out of 70) beekeepers. Out of these, 15 were detected only by ITS2, 10 only by *rbcL* and nine by both markers. Table 3 presents information about the frequency and distribution of the key species. In four samples taken from East Azerbaijan none of the key species were detected. If the beekeepers have given the correct information, these samples were produced in west of Iran. Of the 33 species 18 of the 21 were unique to north, 10 of 13 unique to south/south west and 2 of 2 for west and overall only 3 species (*Oryza sativa*, *Citrus aurantiifolia* and *Citrus maxima*) had overlapping distributions between north and south/south west regions. In two samples only *Oryza sativa* (rice) was found and although rice is mainly cultivated in the north of Iran, broad areas of Fras province in south of Iran also host rice fields. Thus, this plant cannot act as a key species without information on the presence of other key species. The same is true for *Citrus* spp. that are planted mainly in northern provinces but also in Fars. However, from what the beekeepers mentioned, we know that the two samples containing only rice were from the north of Iran.

**Table 3:** The key species, their frequency and occurrence in samples, and their distribution. Occurrence means the number of samples containing the corresponding taxa. Average frequency equals the total number of reads for a taxon divided by occurrence.

Taxa	Occurrence		Average frequency		North	West	South/South-west
	ITS2	*rbcL*	ITS2	*rbcL*			
*Acanthophyllum kurdicum*	25	0	46	0		∗	
*Acer velutinum*	4	0	4	0	∗		
*Actinidia deliciosa*	8	5	201	294	∗		
*Alnus glutinosa*	3	9	22	55	∗		
*Alnus subcordata*	6	0	94	0	∗		
*Arachis hypogaea*	2	2	4	17	∗		
*Camellia sinensis*	4	10	7	200	∗		
*Citrus aurantiifolia*	0	1	0	30	∗		∗
*Citrus maxima*	0	6	0	42	∗		∗
*Cupressus sempervirens*	4	0	8	0	∗		
*Dicyclophora persica*	3	0	42	0			∗
*Diospyros lotus*	12	13	163	2104	∗		
*Eriobotrya japonica*	0	8	0	101	∗		
*Fagonia acerosa*	31	0	94	0			∗
*Fagus orientalis*	2	0	8	0	∗		
*Fraxinus excelsior*	5	3	121	58	∗		
*Glycine max*	0	5	0	40	∗		
*Hypericum androsaemum*	0	2	0	42	∗		
*Ilex aquifolium*	6	10	40	51	∗		
*Levisticum officinale*	2	0	4	0			∗
*Lisaea strigosa*	23	0	58	0		∗	
*Oryza sativa*	32	32	1660	540	∗		∗
*Paliurus spina-christi*	2	3	10	35			∗
*Parrotia persica*	3	0	8	0	∗		
*Pterocarya fraxinifolia*	0	2	0	29	∗		
*Phlomis persica*	4	0	80	0			∗
*Prosopis cineraria*	0	6	0	7591			∗
*Prosopis juliflora*	0	2	0	17			∗
*Rhabdosciadium aucheri*	4	0	22	0			∗
*Rubus idaeus*	0	11	0	771	∗		
*Solanum villosum*	4	0	70	0			∗
*Taxus baccata*	0	9	0	53	∗		
*Tilia platyphyllos*	6	0	57	0	∗		
*Verbascum kermanense*	32	0	66	0			∗

There were also 10 samples containing the mixture from the west and south/south-west elements. However, the dominant key species always correspond to the correct migration route.

The number of key species found for north, south/south-west, and west of Iran is 22, 13 and 2, respectively. In other words, the number of key species found for the north of Iran is 1.5 times the number of species found for the two other regions. This large difference is due to the fact that the climate and accordingly the vegetation in north of Iran is distinct from other regions which are more similar in climate and have many plant species in common (Takhtajan, 1986; Sagheb-Talebi et al., 2014). This is the reason why the mixture of the west and south/south-west elements was found in 10 samples. Also the low number of key species from west may explain why in four samples from this migration category neither of the key species was found.

The determination of geographical origin can be done for the north of Iran even according to the altitudinal gradient. For example, samples that contain cultivated genera such as *Actinidia*, *Camellia, Citrus*, and *Oryza* are produced mainly in areas from coastal plain up to 100 m elevations, whereas native genera such as *Diospyros* (*lotus*)*, Pterocarya, Ilex,* and *Parrotia* are mainly confined to elevations up to 1000 m of northern Alborz Mountains.

The average number of sequences belonging to key species per sample was 117 and 635 for ITS2 and *rbcL*, respectively. Dividing these numbers by the average number of merged sequences per sample for ITS2 (51,032) and *rbcL* (9,445) indicates that sequences from key species comprise on average 0.2 and 6.7 percent of sequences originated from ITS2 and *rbcL*, respectively. The greater value for *rcbL* derived from only four species, otherwise, in general the contribution from key species is very low. Some issues such as PCR bias impede the calculation of the real frequency of a species in the sample based on the number of sequences. However, as the majority of key species show a low contribution to the total merged sequences per sample, it can be deduced that most of the key species are rare inside the honey samples, and thus they may not be seen during the microscopy analysis.

## 4 Conclusion

The multilocus metabarcoding using a combination of ITS2 and *rbcL* is a robust method to locate the geographical origin of honey as it can detect the DNA from rare pollen grains and identify plants down to species level. However, the resolution of determination greatly depends on the plant diversity throughout the study areas and our knowledge of this diversity. Gathering the floristic data, however, is a laborious task and much effort was devoted in this study to select the key plant species.

The successful discrimination of samples originated from the studied migration routes and the substantial difference between the flora of north compared to west and south-west of Iran indicates that metabarcoding can be employed to determine the origin of honey at large geographical scales.

To adapt metabarcoding as a routine method for authentification of geographical origin in commercial labs, two approaches can be taken: first, exploring the key plant species from origins with bad reputation for adulteration and second, analyzing large amounts of honey samples from all main honey producing countries and utilizing machine learning to discriminate between the origins. The first approach needs less samples to be analyzed, however requires extensive literature review to list the key pollen types that are locally endemic or at least distributed in a small area. The second approach does not need deep floristic knowledge of different regions and can reveal more sophisticated trends in frequency of plant species in honey, but it will be very costly. Nevertheless, as resolution and exactness in determination of geographical origin matter, developing the metabarcoding based methods seems to be a worthwhile investment.

## Declarations

### Author contribution statement

E. Khansaritoreh: Performed the experiments, conceived and designed the experiments; Analyzed and interpreted the data; Wrote the paper.

Y. Salmaki, E. Ramezani, T. Akbari Azirani: Analyzed and interpreted the data; Contributed reagents, materials, analysis tools or data; Wrote the paper.

A. Keller: Contributed reagents, materials, analysis tools or data; Wrote the paper.

K. Neumann: Performed the experiments; Contributed reagents, materials, analysis tools or data; Wrote the paper.

K. Alizadeh: Conceived and designed the experiments; Analyzed and interpreted the data; Wrote the paper.

S. Zarre, G. Beckh, H. Behling: Analyzed and interpreted the data; Contributed reagents, materials, analysis tools or data; Wrote the paper.

### Funding statement

This work was supported by 10.13039/100008791Bayer CropScience and Quality Service International GmbH.

### Declaration of interests statement

The authors declare no conflict of interest.

### Additional information

Supplementary content related to this article has been published online at https://drive.google.com/drive/folders/1NIuGzX7LdOu8ru5XrNJtlELpD0laVkbz?usp=sharing.
